# Prevalence of Methylphenidate Misuse in Medical Colleges in Pakistan: A Cross-sectional Study

**DOI:** 10.7759/cureus.5879

**Published:** 2019-10-09

**Authors:** Nismat Javed, Fatimah Ahmed, Sikandar Saeed, Raham Amir, Hadia Khan, Saima P Iqbal

**Affiliations:** 1 Internal Medicine, Shifa College of Medicine, Shifa Tameer-E-Millat University, Islamabad, PAK; 2 Surgery, Shifa College of Medicine, Shifa Tameer-E-Millat University, Islamabad, PAK; 3 Family Medicine, Shifa International Hospitals, Islamabad, PAK

**Keywords:** methylphenidate, substance abuse, pakistan

## Abstract

Objective

To determine the prevalence of nontherapeutic use of methylphenidate as well as to ascertain any benefits, side effects, and other factors associated with this use.

Materials and Methods

A cross-sectional study was conducted in medical colleges in Pakistan using a self-constructed, validated questionnaire. The sample size was calculated using Open Source Statistics for Epidemiological Health software (OpenEpi web-based open-source program, MIT license), and it was determined to be 400. The type of sampling was cluster sampling. Cronbach's alpha was used to assess the internal consistency of the questionnaire, and it was found to be 0.80.

Results

Out of the 400 participants, 197 (49%) were male and 203 (51%) were female. The mean age of the participants was 21.02 (±1.54) years. Most of the participants (84%) reported they usually studied 1-3 hours a day, and 149 participants (37%) reported a score ranging from 70 to 80% in exams. Methylphenidate was admitted to be used by 37 participants. Out of these 37 participants, only 10 participants believed they had attention deficit hyperactivity disorder (ADHD). Participants revealed they used the drug to either cope with the anxiety related to their scores or merely as a personal, recreational choice. Methylphenidate was mostly acquired from peers (68%), and peer pressure was found to be a major factor in its misuse.

Conclusions

Most of the methylphenidate misuse is linked to student underperformance in examinations and an underlying dissatisfaction. The problem is more aggravated when the social influence exerted by other students is taken into account. The side effects of drug usage are found to outweigh the benefits that have been reported.

## Introduction

Substance abuse is defined as an erratic behavior pattern of substance use leading to impairment and, ultimately, tolerance and withdrawal, usually occurring within a twelve-month period of the starting of the abuse [1]. According to the UN World Drug Report 2017, about 271 million people have been victims of illicit drug use the world over [2]. Annual estimates in Pakistan suggest that there are 6.7 million drug users in the country, and most of these users belong to the economically productive age group of 25-39 years [3].

One of the substance-abuse categories is medication abuse. The number of people being prescribed medications is increasing and, therefore, with the increased availability of drugs, the potential for abuse has also increased. According to recent global statistics, 1.3 million teenagers have reported using Ritalin or Adderall, common brand names under which methylphenidate is sold, in the last year [4].

In 2015, a researcher writing for one of the College Life Study publications quoted the data provided by the American College Health Association that indicated that the incidence of stimulant use in college students stands at a remarkable 10.0% [5]. This behavior is mostly seen as a coping mechanism for students who are under enormous pressure to academically perform well at universities. Some students turn to illicit substance abuse as a stimulant to help them catch up with their studies, which lets them compensate for the lost time they spent on other activities [6]. A study of third-year university students conducted in New Zealand in 2015 reported a rate of 6.6% for the use of cognitive enhancers, including methylphenidate [7]. More recently, a study in South Africa reported that 17.2% of undergraduate students at one university had used methylphenidate with only 2.9% among them being diagnosed with ADHD [8].

Evidence suggests that medical students may be especially prone to medication abuse. With the demanding changes brought about in various integrated curriculum, these students often fear about underperforming in academics and may feel the pressure to rectify it [9]. Other stimuli included weak parental influence [10], economic uncertainty, peer pressure, and stress [11]. In 2015, another study among Israeli medical students reported that 13.5% admitted to methylphenidate use during the past year [12]. Approximately 10.0% of students reported ever being diagnosed with ADHD. A recent study among second-year and fourth-year medical students at one South African university found 13.1% (33/251) reporting substance use for non-medical purposes in the preceding year, with only 2% reporting an ADHD diagnosis [13].

Students using methylphenidate can experience numerous side effects, such as hallucinations, anxiety, dry mouth, and visual disturbances [14]. Withdrawal symptoms can include fatigue, disturbed sleeping patterns, and depression, while use in high doses can result in cardiovascular failure or lethal seizures [15].

The objective of this study was to determine the frequency of methylphenidate abuse among medical students in Pakistan and to determine the associated stimuli that caused them to resort to such prescription drugs.

## Materials and methods

We conducted a cross-sectional study of students from medical colleges in Islamabad and Rawalpindi from June 2018 to March 2019. The sample size was calculated using Open Source for Epidemiological Statistics software (OpenEpi web-based open-source program, MIT license) and was found to be 400 [16]. The students were verbally informed about the study, and those who gave their consent were included. The sampling method used was cluster sampling. All medical colleges of Islamabad and Rawalpindi were listed. The administration of each college was approached for the research, and two medical institutes gave their consent. All the students who had been part of the institution for more than six months were included. Students who were not present on the day of the study were not included.

The data was collected through a self-constructed questionnaire. The questionnaire was assessed for reliability using a pilot study consisting of 40 medical students who were selected randomly and from the results of this study. The questionnaire was not changed and was administered shortly afterward. Cronbach's alpha was used to assess the internal consistency of the questionnaire, and it was found to be 0.79.

The questionnaire comprised 16 questions of which three were open-ended and 13 were close-ended. The questionnaire assessed the students’ percentage score in their modules and the associated emotions with the scores. The participants were also asked the coping mechanisms associated with the emotions that they displayed pertaining to their scores. The questionnaire used a scale to enable the participants to indicate their percentage scores. The scale in question was similar to the one used by universities to gauge attendance. The questionnaire also enabled the participants to report if there had been any prior diagnosis of ADHD and the frequency of drug usage if any. The participants were also asked to report the sources of the drugs and also the influences that caused them to use such drugs.

The questionnaire was distributed to medical students of all five academic years in different medical colleges. The participants were given 30-45 minutes to complete the entire questionnaire. A total of 800 questionnaires were distributed, of which 400 questionnaires (50%) were completed. After the completion of the study, a 20-minute session was conducted to address any queries from the participants.

The data obtained was analyzed on IBM's Statistical Package for the Social Sciences (SPSS) version 21 (IBM, Armonk, NY). Descriptive statistics were used for the analysis and description of the data. Frequencies and percentages were calculated for participant distribution according to gender.

## Results

Out of the 400 participants, 197 (49%) were male and 203 (51%) were female. The mean age of the participants was 21.02 (±1.54) years. The distribution of participants is shown here (Table 1).

**Table 1:** Distribution of participants according to hours spent on studying

Hours spent on studying	Frequency, n
0-3	336 (84%)
4-7	58 (14%)
8-12	4 (1%)
>12	2 (1%)

Out of the 400 participants, 390 (98%) had not been diagnosed with ADHD, and 10 (2%) might have been diagnosed with ADHD. No participant reported an objective confirmation of the diagnosis by a health-care professional. The distribution according to scores in exams is shown here (Table 2). 

**Table 2:** Distribution according to percentage score in exams

Percentage score in exams	Frequency, n
<50	6 (1%)
>50 and <60	36 (9%)
>60 and <70	143 (36%)
>70 and <80	149 (37%)
>80	66 (17%)

**Figure 1:**
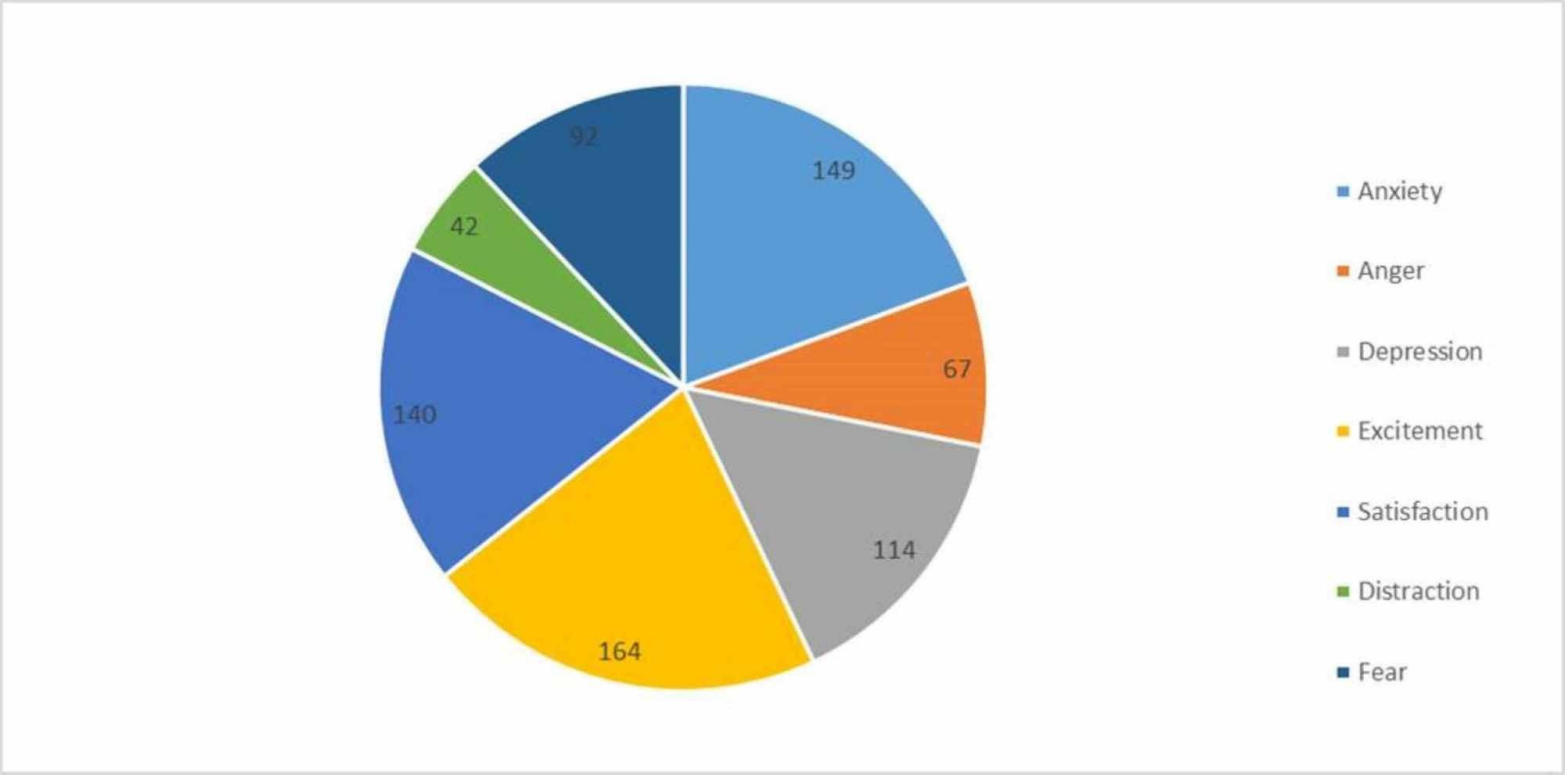
Participants’ emotions about their academic performance

The participants were also asked to describe their feelings pertaining to the scores they had been achieving. The results are shown below (Figure 1).

Out of the 400 participants, 5% reported that the issue of drug usage was mostly tackled by social media, while 37 (9%) participants reported using methylphenidate at least once. As many as 68% of the participants who used drugs reported that peers were the most common source for acquiring drugs. Medical officers (32%) were the second most common source. These participants also reported that peer pressure was the main stimulus for their continued drug use.

**Figure 2:**
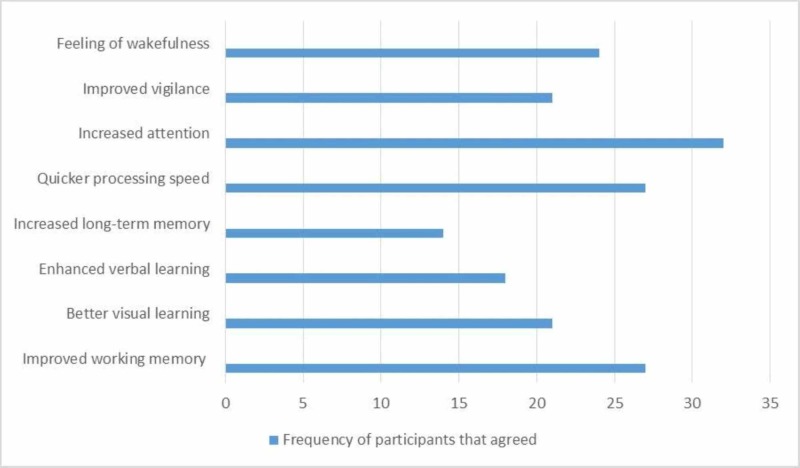
Beneficial effects of drugs according to users

The beneficial effects of using methylphenidate as reported by 37 participants who had used the drug are shown here (Figure 2). 

The same number of participants (51%) reported that they felt the need to use the drug prior to taking a test. Some participants (32%) reported that they felt the urge to use the drug again after less than 1 hour of taking a test.

**Figure 3:**
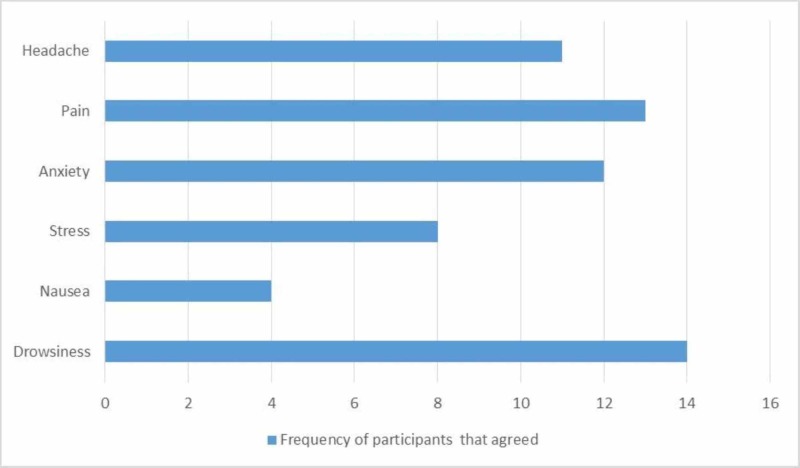
Side effects of drug usage

The participants also reported the side effects they experienced after taking the drug. In this case, the participants were given the chance to indicate more than one option as provided in the questionnaire. The results are shown below (Figure 3).

There was a difference in the levels of stress as a side effect of methylphenidate usage among both genders, and this was found to be significant when tested using chi-square [probability value (p): <0.05). Out of the 37 participants, 20 (54%) reported an improvement in exam scores. While 3 (1%) reported no improvement, 14 (35%) were unsure of their improvement.

## Discussion

This was the first study conducted consisting of medical students from Islamabad and Rawalpindi that described the prevalence of methylphenidate misuse and the associated epidemiological, social, psychological, and, possibly, biological characteristics. As many as 400 medical students from various medical colleges participated in the study, and the ratio of female-to-male participants was 1:1. The findings of our study were close to those from a similar study done in Iran [17]. In our study, the mean age of participants was 21.02 (±1.54) years, which was much lower than the mean age reported in another Iranian study [23.02 (±0.29) years] [18]. There was no significant difference in drug usage among both genders, and this data is similar to the findings of other studies, which revealed 3% of Asian women to have used a stimulant drug compared to 3% of Asian men [19]. The results of our study showed that 98% of the participants had not been diagnosed with ADHD, and 37 participants reported using methylphenidate. This distribution was higher for our study when compared to a 2009 interview-style study [20].

An interesting finding of our study was related to participants' emotions about their performance. Anxiety and satisfaction had similar distribution among the responses, indicating that the choice of misusing the drug is not only related to academic performance but can be a merely personal, recreational choice as well [21]. The possible reasons for such a choice were beyond the scope of this study. Peer pressure was shown to be the primary stimulus leading to drug abuse [22]. Easy access to such drugs makes the entire process simpler, particularly when the peers in question are also part of the medical profession [22]. Anxiety, however, can be explained, as students’ academic issues ultimately trigger a vicious cycle of depression that further aggravates their condition [23].

Improved attention and increased level of wakefulness were some of the beneficial effects reported by the participants in our study. This is a peculiar finding because complex cognitive functions were previously thought to be unaffected by this type of drug abuse. A study conducted in 2016 provides a possible explanation as it states that methylphenidate activates the high-attention areas, particularly the cerebellum, temporal lobe, and occipital lobe [24].

Common side effects that our study found included drowsiness and generalized aches. The sensation of pain experienced by the participants afterward was a new finding. Our study also reported rebound stress as a significant side effect of the drug abuse, which is the least frequent side effect reported by another review-based study [25]. Headache was also reported by many participants. This side effect is probably the most chronic one as it was reported by many of the participants [26].

An improvement in their exam scores was also reported by some participants. This improvement in score can be attributed to the reflection of complex decision-making processes, but with a relaxed time frame. Students who might have chronically used methylphenidate and started at an early age did not report any improvement in their scores as cognitive processes are relatively slower [27,28].

There are some limitations to the study. This is a cross-sectional study and, apart from a chi-square test, no other statistical analysis could be performed. The study only includes two cities of Pakistan and must be expanded to include other regions as well, especially the regions labeled as high risk for drug misuse. This study does not extensively explore the reasons for a participant opting for drug abuse, regardless of academic performance or environment.

## Conclusions

Methylphenidate misuse has become a major problem for the vulnerable segment of the population in Pakistan, particularly medical students. Increased attention and wakefulness that the drug generates may give the impression that the drug can help the students in excelling in their studies. However, long-lasting rebound stress is a major disadvantage and is responsible for triggering a vicious cycle of drug dependence. The side effects of drug use are found to outweigh the benefits considerably. Moreover, concerns regarding academic performance are not the only reason for drug abuse, and the "personal reasons" cited by many of the participants in our study must be further explored to get a clearer picture of the phenomenon.
